# Lipopolysaccharide acting via toll-like receptor 4 transactivates the TGF-β receptor in vascular smooth muscle cells

**DOI:** 10.1007/s00018-022-04159-8

**Published:** 2022-02-05

**Authors:** Rizwana Afroz, Hirushi Kumarapperuma, Quang V. N. Nguyen, Raafat Mohamed, Peter J. Little, Danielle Kamato

**Affiliations:** 1 School of Pharmacy Australia Centre of Excellence, The University of Queensland, Woolloongabba, QLD 4102 Australia; 2 Department of Pharmacy, Xinhua College of Sun Yat-Sen University, Tianhe District, Guangzhou, 510520 China; 3 Sunshine Coast Health Institute, University of the Sunshine Coast, Birtinya, QLD 4575 Australia; 4 Centre for Cancer Cell Biology and Drug Discovery, Griffith Institute for Drug Discovery, Griffith University, Nathan, Brisbane, QLD 4111 Australia; 5 Department of Basic Sciences, College of Dentistry, University of Mosul, Mosul, Iraq

**Keywords:** Smad, Transforming growth factor beta, Transactivation dependent

## Abstract

Toll-like receptors (TLRs) recognise pathogen‑associated molecular patterns, which allow the detection of microbial infection by host cells. Bacterial-derived toxin lipopolysaccharide activates TLR4 and leads to the activation of the Smad2 transcription factor. The phosphorylation of the Smad2 transcription factor is the result of the activation of the transforming growth factor-β receptor 1 (TGFBR1). Therefore, we sought to investigate LPS via TLR4-mediated Smad2 carboxy terminal phosphorylation dependent on the transactivation of the TGFBR1. The in vitro model used human aortic vascular smooth muscle cells to assess the implications of TLR4 transactivation of the TGFBR1 in vascular pathophysiology. We show that LPS-mediated Smad2 carboxy terminal phosphorylation is inhibited in the presence of TGFBR1 inhibitor, SB431542. Treatment with MyD88 and TRIF pathway antagonists does not affect LPS-mediated phosphorylation of Smad2 carboxy terminal; however, LPS-mediated Smad2 phosphorylation was inhibited in the presence of MMP inhibitor, GM6001, and unaffected in the presence of ROCK inhibitor Y27632 or ROS/NOX inhibitor DPI. LPS via transactivation of the TGFBR1 stimulates PAI-1 mRNA expression. TLRs are first in line to respond to exogenous invading substances and endogenous molecules; our findings characterise a novel signalling pathway in the context of cell biology. Identifying TLR transactivation of the TGFBR1 may provide future insight into the detrimental implications of pathogens in pathophysiology.

## Introduction

Toll-like receptors (TLRs) play a critical role in bridging innate and acquired immune responses [1]. TLRs are activated by both pathogen‑associated molecular patterns (PAMPs) and damage‑associated molecular patterns (DAMPs). These receptors belong to the pattern recognition (PRR) family and underpin the pathology of numerous inflammation-related diseases, including cardiovascular disease (CVDs), diabetes and several types of cancers. The first cloned and characterised human TLR was TLR4, which is activated by lipid A, the biologically active constituent of bacterial endotoxin, lipopolysaccharide (LPS) [2]. TLRs, specifically TLR4, have an established role in multiple cardiovascular pathophysiologies [3–5], including our recent work demonstrating that LPS drives proteoglycan modifications as an initiating step in atherosclerosis [6]. LPS signalling utilises intracellular adapter molecules such as myeloid differentiation factor-88 (MyD88) and TIR domain-containing adaptor protein (TRIP) [7]. The activation of the TLR-linked adapter molecules results in the consequent activation of downstream transcription factors NF-κB and AP-1. Emerging data have shown that TLR4 can also activate the Smad transcription factor [8–13]. Therefore, we sought to investigate the mechanism of TLR-mediated Smad signalling in human vascular smooth muscle cells (VSMCs).

Direct or canonical TGF-β signalling commences upon the binding of the TGF-β to the TGF receptor type II, which leads to phosphorylation and recruitment of the TGF-β receptor type I (TGFBR1) to form a quaternary TGF-β receptor complex that leads to the phosphorylation of the Smad transcription factor [14]. TGF-β is produced in a ‘latent’ form in cells and requires activation before eliciting cellular effects. The activation of the latent TGF-β is achieved through various mechanisms, involving integrins, proteases and reactive oxygen species (ROS) [15, 16]. Our group identified that G protein-coupled receptors (GPCR) such as thrombin [17–20], endothelin [21, 22] and lysophosphatidic acid [23, 24] via specific biochemical mechanisms could transactivate the TGFBR1 leading to the phosphorylation of Smad2. Transactivation-dependent signalling involves cell surface receptor transactivation of a second cell surface receptor occurring in the absence of transcription and translation of intermediate products [25, 26]. The engagement of transactivation signalling considerably expands the range of cellular functions attributable to the first or index cell surface receptor. As an example of the quantitative contribution of transactivation signalling to the primary receptor signalling response, we have demonstrated that transactivation-dependent signalling accounts for 50% of the total pool of genes regulated by the cognate receptor ligand and thrombin activation of protease-activated receptor (PAR)-1 [27].

The mechanisms involved in GPCR transactivation of the TGFBR1 have been characterised in detail. We have found that in VSMCs, thrombin [17–19] and lysophosphatidic acid [23, 24] transactivation of the TGFBR1 occurs via cytoskeletal rearrangement involving Rho‑associated protein kinase (ROCK)‑signalling, which leads to the activation of integrin proteins on the cell surface. Once activated, cell surface integrin induces the activation of the large latent complex (LLC) that releases TGF‑β for activation of TGFBR1 [19, 28]. The signalling paradigm described by our group was observed in several other models including lung epithelial cells [29], rat VSMCs [30] and airway smooth muscle cells [31]. The TGFBR1 can be activated by matrix metalloproteinases (MMPs) that can cleave and liberate the TGF-β ligand from the complex and activate downstream TGF-β signalling [16]. The role of MMPs was assessed in GPCR transactivation of the TGFBR1 and we showed surprisingly that thrombin transactivation of the TGFBR1 was not dependent on MMPs, noting that MMP involvement in transactivation of the epidermal growth factor (EGF) receptor was one of the first documented manifestations of transactivation signalling [32].

The link between infection and cardiovascular disease has been identified; however, the mechanisms have not been fully elucidated. The identification that pathogen-sensing receptors can communicate and activate secondary cell surface receptors can explain the consequences of TLRs on several vascular pathophysiologies. TGF‑β and its cognate receptor correlate with the progression of vascular diseases, including atherosclerosis [18, 33–35]. TGFBR signalling activates multiple signalling intermediates that control the transcription of genes involved in vascular pathophysiology [36]. One such TGF‑β target gene encodes plasminogen activator inhibitor type 1 (PAI‑1), a proteinase inhibitor essential for homeostasis and preclusion of induction of bleeding diathesis [37]. Increased expression of PAI–1 mRNA has been identified in various cell types, primarily localised around the base of atherosclerosis plaque [38, 39]. Administration of a PAI–1 inhibitor decreases atherosclerosis in a rodent model of obesity and the metabolic syndrome [40]. Mice express a stable form of PAI‑1 in the blood that can contribute to the development of coronary artery clots without typical precursor atherosclerosis [37, 41]. Therefore, we have considered PAI‑1 expression a functional readout of pathophysiologically interesting TLR4/TGFBR interactions in VSMCs.

In this study, we investigated the role of LPS and TLR4 in activating TGFBR, which was assessed by the phosphorylation of transcription factor Smad2 in the carboxy terminus. We characterised the signalling mechanisms by which TLR4 transactivates TGFBR in VSMCs and measured PAI‑1 expression level as a functional readout of this signalling pathway.

## Methods and materials

### Materials

Human aortic-vascular smooth muscle cells (HA-VSMCs) (ATCC® CRL-1999) were purchased from In Vitro Technologies Life Sciences (VIC, Aus). LPS (rough strains from *Salmonella enterica* serotype minnesota Re 595 (Cat #L9746)), SB431542, AG1478, SB202190, UO126 and SP600125 were purchased from Sigma-Aldrich (MO, USA). GM6001, pyridoxatin and Ab1421180 were purchased from Abcam (VIC, Australia). Human recombinant transforming growth factorβ1(TGFβ1), antiphosphoSmad2 (Ser465/467), antirabbit imunoglobulinG (IgG) horseradish peroxidase (HRP) and glyceraldehyde 3-phosphate dehydrogenase (GAPDH) rabbit monoclonal IgG antibody (HRP conjugate) were purchased from Australian Bioresearch (WA, AU). Primers to 18S and PAI-1were purchased from Qiagen (VIC, AUS).

### Cell culture

HA-VSMCs (passage 5–9) were grown in Ham’s F-12 K (Kaighn’s) medium supplemented with 10% FBS (v/v) and 1% penicillin streptomycin (v/v) at 37 °C in 5% CO_2_. HA-VSMCs were seeded in 60 mm dishes for experimentation. Cells were grown to confluence and rendered quiescent condition with Ham’s F12K Ham (0.1% FBS (v/v)) for 48 h prior the experiment.

### Quantitative real-time polymerase chain reaction

The mRNA expression level of PAI-1 was determined by qRT-PCR. Total RNA was extracted from treated cells using RNeasy Mini kit (Qiagen). RNA concentration and purity were checked by spectrophotometry using Nanodrop 2000 spectrophotometer (Thermo Fisher Scientific). First-strand cDNA was synthesised from 1000 ng RNA using the Quantitect® reverse transcription kit (Qiagen). qRT-PCR was performed using Qiagen Rotor gene Q and QuantiNova™ Sybr® green PCR kit (Qiagen). The data were normalised against 18S using the delta delta cycle-threshold (ΔΔCt) method.

### Western blotting

Protein isolated from treated cells was separated and transferred onto polyvinylidene difluoride membranes. Membranes were blocked for 60 min at room temperature with 5% BSA and probed with anti-phospho-Smad2 (Ser465/467) (1:1000 dilution) overnight at 4°C, followed by rabbit immunoglobulin-G (IgG)- HRP secondary antibody (1:1000 dilution) for 60 min at room temperature. The membranes were stripped and probed with GAPDH HRP conjugated monoclonal antibody (1:4000 dilution) to determine equal loading. Enhanced chemiluminescence (ECL) was used to visualise proteins of interest. Blots were imaged with Bio-Rad gel documentation system. Band density was measured by Image Lab imaging software (version 5.2.1). The protein of interest was normalised against housekeeping protein GAPDH.

### Assessment of MMP activity by gelatine zymography

Confluent HA-VSMCs were washed with serum-free media (0% FBS (v/v)) and then incubated for 48 h. Following treatment, conditioned media were collected. Equal amounts of conditioned media were loaded on a 10% SDS polyacrylamide gel co-polymerised with 1 mg/mL of gelatine and electrophoresed at 120 V for 3 h on ice [42]. The gel was incubated in wash buffer (Triton X-100 2.5% (v/v), 50 mM Tris–HCl pH 7.5 (v/v), 5 mM CaCl_2_ (v/v))  30 mins twice  with gentle agitation. The gel was rinsed with incubation buffer (Triton X-100 1% (v/v), 50 mM Tris–HCl pH 7.5 (v/v), 5 mM CaCl_2_ (v/v)) for 10 min. The gel was incubated for 24 h (at 37 °C) in fresh incubation buffer and then stained with Coomassie brilliant blue R250 staining solution for 1 h, followed by soaking in de-staining solution (methanol 25% (v/v), acetic acid 10% (v/v) and H_2_O 65% (v/v) until clear white bands against a dark blue background appeared. The bands were detected in a ChemiDoc system and MMP activity was quantified by integrative densitometry using ImageJ (v1.52) software.

### Statistical analysis

Data were normalised and presented as the mean ± standard error of the mean (SEM). A one-way analysis of variance (ANOVA) was used to analyse statistical significance followed by least significant difference (LSD) post hoc analysis. Results were significant when the probability was less than 0.05 (*p < 0.05) and 0.01 (**p < 0.01).

## Results

### LPS-mediated Smad2 carboxy terminal phosphorylation is dependent on TGFBR1 activation

**Fig. 1:**
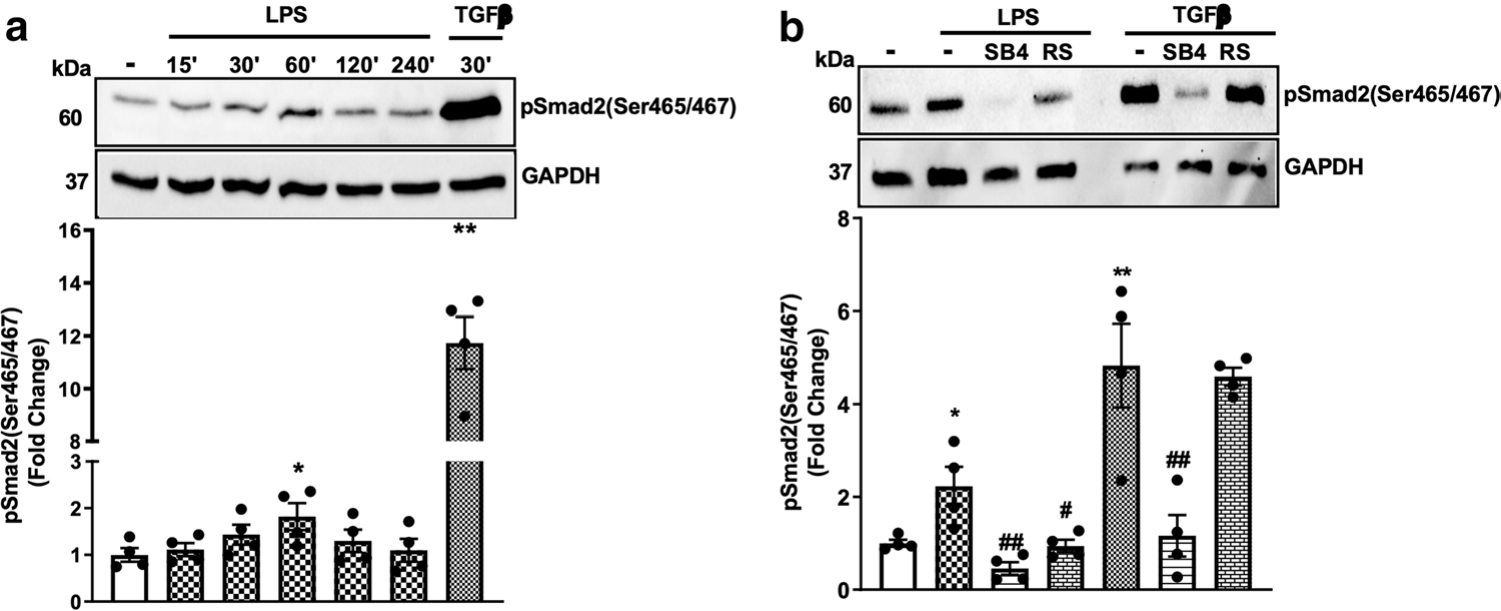
LPS stimulates Smad2 carboxy terminal phosphorylation via transactivation of the TGFBR1. HA-VSMCs were treated with **(a)** LPS (100 ng/mL) for 0–240 min and TG-Fβ (2 ng/mL) for 30 min or **(b)** LPS (100 ng/ml) for 60 min or TGFβ (2 ng/mL) for 30 min in the presence and absence of TGFBR1 antagonist SB431542 (SB4) (3 μM) or the TLR4 inhibitor LPS-RS (RS) (100 ng/mL). Membranes were probed with monoclonal antiphosphoSmad2 (Ser465/467) (1:1000 dilution), followed by HRP-labelled antirabbit IgG secondary (1:1000 dilution), then stripped and probed with HRP-conjugated anti-GAPDH (1:4000 dilution). Histograms represent band density expressed as fold change compared to no treatment control from four independent experiments. *p < 0.05 agonist versus untreated control and ##*p* < 0.01 agonist versus antagonist using one-way ANOVA followed by LSD post hoc test

LPS stimulates Smad2 carboxy-terminal phosphorylation (Ser465/467) and its nuclear translocation in cultured hepatic stellate cells (HSC‑T6) [43]. We have recently reported that LPS stimulates Smad2 linker region phosphorylation in HA-VSMCs [6]; however, the role of LPS-mediated Smad2 carboxy-terminal phosphorylation in HA‑VSMCs remains to be elucidated. HA-VSMCs were treated with LPS in a time-dependent manner (0–240 min), and TGF‑β was used as a control. Treatment with LPS for 30 min caused a 1.4-fold increase in the phospho-Smad2 carboxy terminal (Fig. 1a) with a peak stimulation of 1.8-fold observed at 60 min. TGF-β, used as a control, resulted in an 11-fold increase in phospho-Smad2 carboxy terminal.

Canonical Smad2 carboxyl-terminal phosphorylation occurs as a direct outcome of the activation of the TGFBR1 [44]. To assess whether LPS signalling via its respective TLR could transactivate the TGFBR1, we utilised a pharmacological approach using the TLR4 antagonist, LPS-RS [45–47], and TGFBR1 antagonist, SB431542 [48]. LPS treatment of HA-VSMCs resulted in a 2.2-fold increase in phospho-Smad2 carboxy terminal (Fig. 1b) that was inhibited in the presence of SB431542. LPS-RS used as a control and completely inhibited LPS-mediated Smad2 phosphorylation. The cells were treated with TGF‑β in the presence and absence of receptor antagonists, and as expected TGF-β-mediated phospho-Smad2 carboxy terminal was completely inhibited in the presence of SB431542 and unaffected in the presence of TLR4 antagonist (Fig. 1b). These findings demonstrate that LPS via its cognate receptor, TLR4 transactivates the TGFBR1 leading to phospho-Smad2 carboxy terminal. These findings highlight the existence of TLR4 transactivation of the TGFBR1 in HA‑VSMCS.

**Fig. 2:**
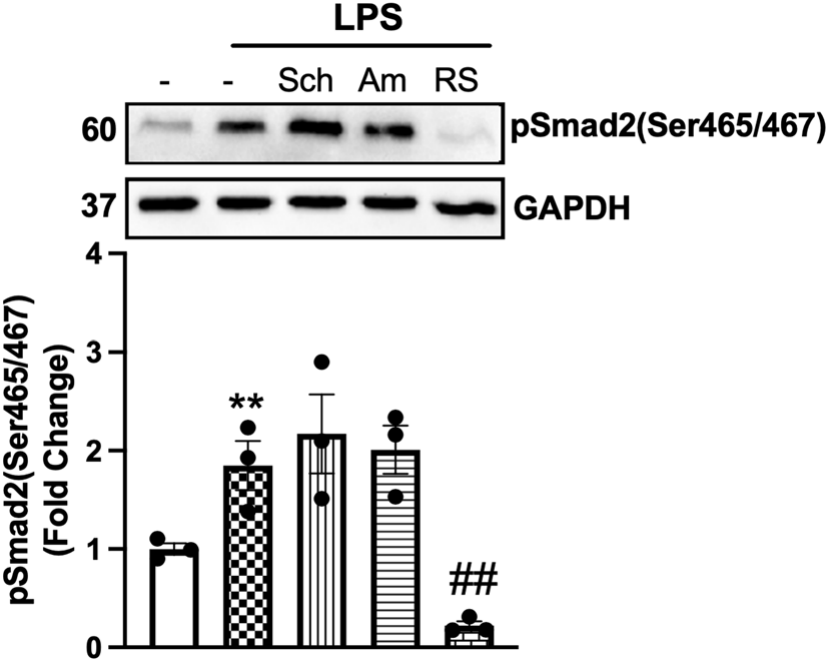
TLR4 transactivation of the TGFBR1 is independent of MyD88 and TRIF-dependent pathways. HA-VSMCs were pre-incubated with schaftoside (Sch) (10 µM), amlexanox (Am) (10 µM) or LPS-RS (RS) (100 ng/mL), followed by LPS (100 ng/ml) for 60 min. Membranes were probed with antiphosphoSmad2 (Ser465/467), followed by the HRP-labelled antirabbit IgG secondary, then stripped and probed with HRP-conjugated anti-GAPDH. Histograms represent band density expressed as fold change compared to no treatment control from three independent experiments. **p < 0.01 agonist versus untreated control and ##*p* < 0.01 agonist versus antagonist using one-way ANOVA followed by LSD post hoc test

### TLR4 transactivation of TGFBR1 is independent of MyD88 and TRIF pathways

TLR signalling pathways are classified into two distinct types: the myeloid differentiation primary response protein-88 (MyD88) and the TIR domain-containing adaptor-inducing IFN (TRIF)-dependent pathways. We asked whether TLR4 transactivation of the TGFBR1 was occurring via MyD88 or TRIF-dependent pathways (Fig. 2). Schaftoside was utilised as an inhibitor of MyD88 signalling, and amlexanox was exploited as a specific inhibitor of the TBK1-inhibiting TRIF-dependent signalling pathways. LPS-mediated phosphorylation of Smad2 carboxy terminal was unaffected in schaftoside- and amlexanox-treated cells; however, it was abolished entirely in the presence TLR4 antagonist LPS-RS. This demonstrates that the LPS-mediated transactivation of the TGFBR1 is independent of MyD88 and TRIF pathways. Therefore, we aimed to delineate the underlying mechanisms of TLR4 transactivation of the TGFBR1.

### TLR4 activates matrix metalloproteinases leading to phosphorylation of Smad2 carboxy terminal

**Fig. 3:**
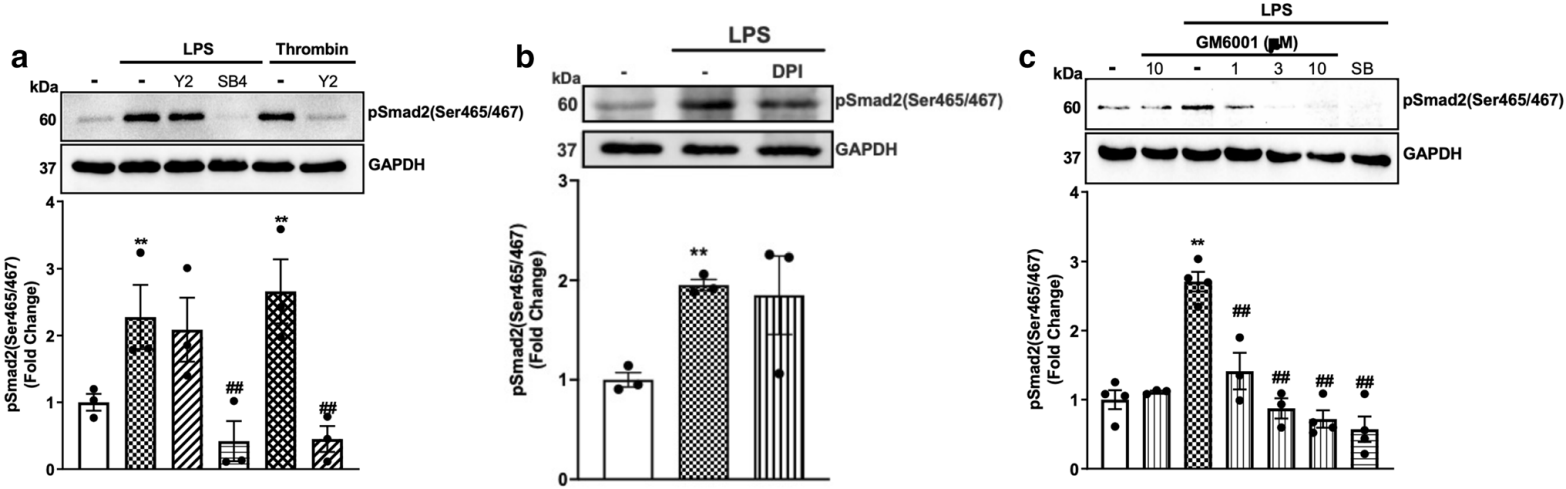
TLR4 transactivation of TGFBR1 is dependent on MMPs: HA-VSMCs were treated with **(a)** LPS (100 ng/mL) and thrombin (10 units/mL) for 60 min in the presence and absence of ROCK inhibitor, Y27632 (Y2) (10 µM) or SB431542 (SB4) (3 μM) (**b)** ROS inhibitor, diphenyleneiodonium (DPI) or **(c)** preincubated with MMP inhibitor GM6001 (1-10 µM) for 30 min followed by LPS (100 ng/mL) for 60 min. Membranes were probed with antiphosphoSmad2 (Ser465/467), followed by the HRP-labelled antirabbit IgG secondary, then stripped and probed with HRP-conjugated anti-GAPDH. Histograms represent band density expressed as fold change compared to no treatment control from four independent experiments. **p < 0.01 agonist versus untreated control and ##*p* < 0.01 agonist versus antagonist using one-way ANOVA followed by LSD post hoc test

We have recently demonstrated that in HA-VSMCs, GPCR transactivation of the TGFBR1 relies on cytoskeletal rearrangement, which activates Rho/ROCK and integrin-dependent signalling leading to conformational changes in the TGF-β complex and the activation of the TGFBR1 [23, 24, 32, 49, 50]. To study if LPS transactivation of the TGFBR1 was dependent on ROCK signalling, we used the potent and selective ROCK inhibitor Y27632 (Fig. 3a). Treatment with LPS resulted in a 2.3-fold increase in phospho-Smad2 carboxy terminal that was unaffected in the presence of the ROCK inhibitor. We used the TGFBR1 antagonist as a control, and as expected, LPS-mediated Smad2 phosphorylation was completely inhibited. We have shown that in VSMCs thrombin-mediated Smad2 phosphorylation was dependent on the Rho/ROCK pathways [32, 49]; therefore, we treated cells with thrombin in the presence and absence of Y27632 as a control. Treatment with thrombin stimulated Smad2 phosphorylation to 2.6-fold, which was completely inhibited in the presence of Y27632. These results demonstrate that LPS-mediated transactivation of the TGFBR1 leading to Smad2 phosphorylation in these cells was not dependent on Rho/ROCK pathways.

The release of the latent TGF-β is triggered by oxidation of the large latent complex which causes conformational changes leading to the release of the TGF-β ligand [51]. To investigate the role of ROS/NOX in TLR4 transactivation of the TGFBR1, we utilised broad-spectrum ROS/NOX inhibitor diphenyleneiodonium (DPI) (Fig. 3b) [50]. LPS-mediated Smad2 phosphorylation was unaffected in the presence of DPI which demonstrates that LPS-mediated transactivation of the TGFBR1 is independent of the ROS/NOX-dependent pathways.

TGF-β can be cleaved and liberated from the large latent complex by proteolytic cleavage of MMPs [52, 53]. To investigate whether LPS-mediated phosphorylation of Smad2 occurs via proteolytic activation of TGFBR1, we utilised the broad-spectrum MMP inhibitor, GM6001 (Fig. 3c). LPS treatment of VSMCs increased phospho-Smad2 carboxy terminal to 2.7‑fold compared to the non‑treated control. This response was inhibited in the presence of GM6001 (1–10 µM) with a complete inhibition observed with 3 µM GM6001. HA‑VSMCs pre‑treated with SB431542 also showed complete inhibition of LPS-mediated phosphorylation of Smad2 carboxy-terminal. These results demonstrate that TLR4 signals via MMPs to activate TGFBR1, which leads to Smad2 carboxy-terminal phosphorylation.

### MMP2, but not MMP9, is involved in LPS stimulation of phospho-Smad2 carboxy terminal

**Fig. 4:**
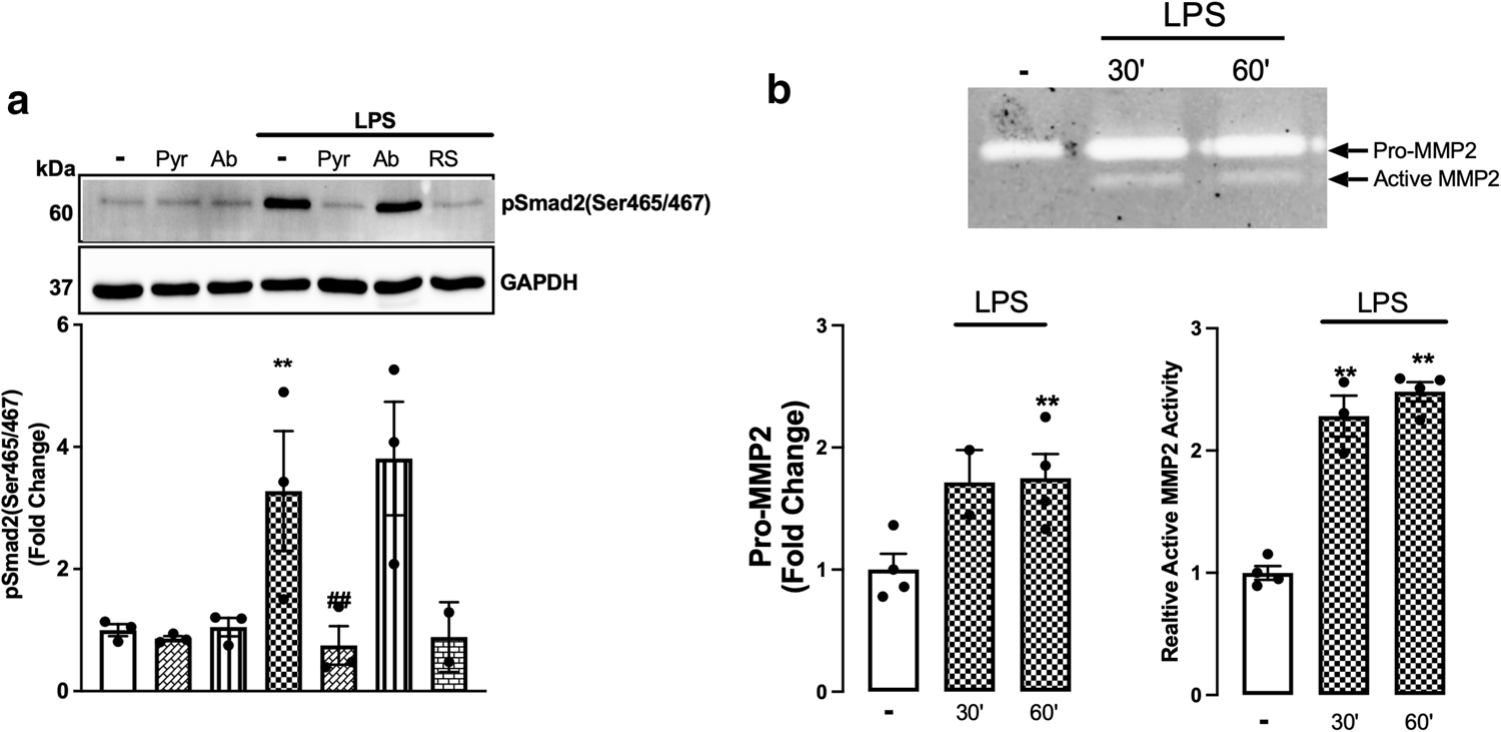
LPS-mediated Smad2 carboxyl terminal phosphorylation is dependent on MMP2 activation. HA-VSMCs were preincubated with **(a)** pyridoxatin (10 µM), Ab1421180 (10 µM) or LPS-RS (100 ng/mL) for 30 min, followed by treatment with LPS (100 ng/mL) for 60 min (**b**) or treated with LPS (100 ng/ml) 30–60 min. **(a)** Membranes were probed with antiphosphoSmad2 (Ser465/467), followed by HRP-labelled antirabbit IgG secondary, then stripped and probed with HRP-conjugated anti-GAPDH. **(b)** The gelatinase activity was assessed from spent media; transparent bands were detected against a background of Coomassie brilliant blue-stained gel. Histograms represent band density expressed as fold change compared to basal from four independent experiments. **p < 0.01 agonist versus untreated control and ##*p* < 0.01 agonist versus antagonist using one-way ANOVA followed by LSD post hoc test

We have identified above that MMPs are involved in LPS transactivation of the TGF-β receptor. Specifically, MMP2, 3, 9, 13 and 14 are involved in the release of TGF-β from the TGF-β complex. LPS increases the activity of MMP2 and MMP9 in rat aortic VSMCs [54]. We sought to investigate the role of MMP2 and MMP9 in TLR4-mediated transactivation of the TGFBR1 using a pharmacological and biochemical approach (Fig. 4a). HA-VSMCs were treated with LPS with MMP2-specific inhibitor, pyridoxatin, or MMP9-specific inhibitor, Ab1421180. Treatment with LPS increased phosphorylation of Smad2 by 3.3-fold that was completely inhibited in the presence of pyridoxatin and unaffected by the presence of AB1421180. We thus demonstrate that LPS signalling is via MMP2-dependent pathways to stimulate phospho-Smad2 in HA-VSMCs.

We next investigated whether LPS-mediated MMP2 secretion from HA-VSMCs (Fig. 4b). LPS treatment of HA-VSMCs resulted in a 1.7-fold increase in the secretion of pro-MMP2, and a 2.4-fold increase in the secretion of active MMP2 was observed over a 60 min treatment. These findings demonstrate that LPS stimulated MMP2 activity in HA-VSMCs, which endorses the involvement of MMPs in TLR4 transactivation of TGFBR1.

### p38 via MMP2 is involved in TLR4 transactivation of TGFBR1 in VSMCs

**Fig. 5:**
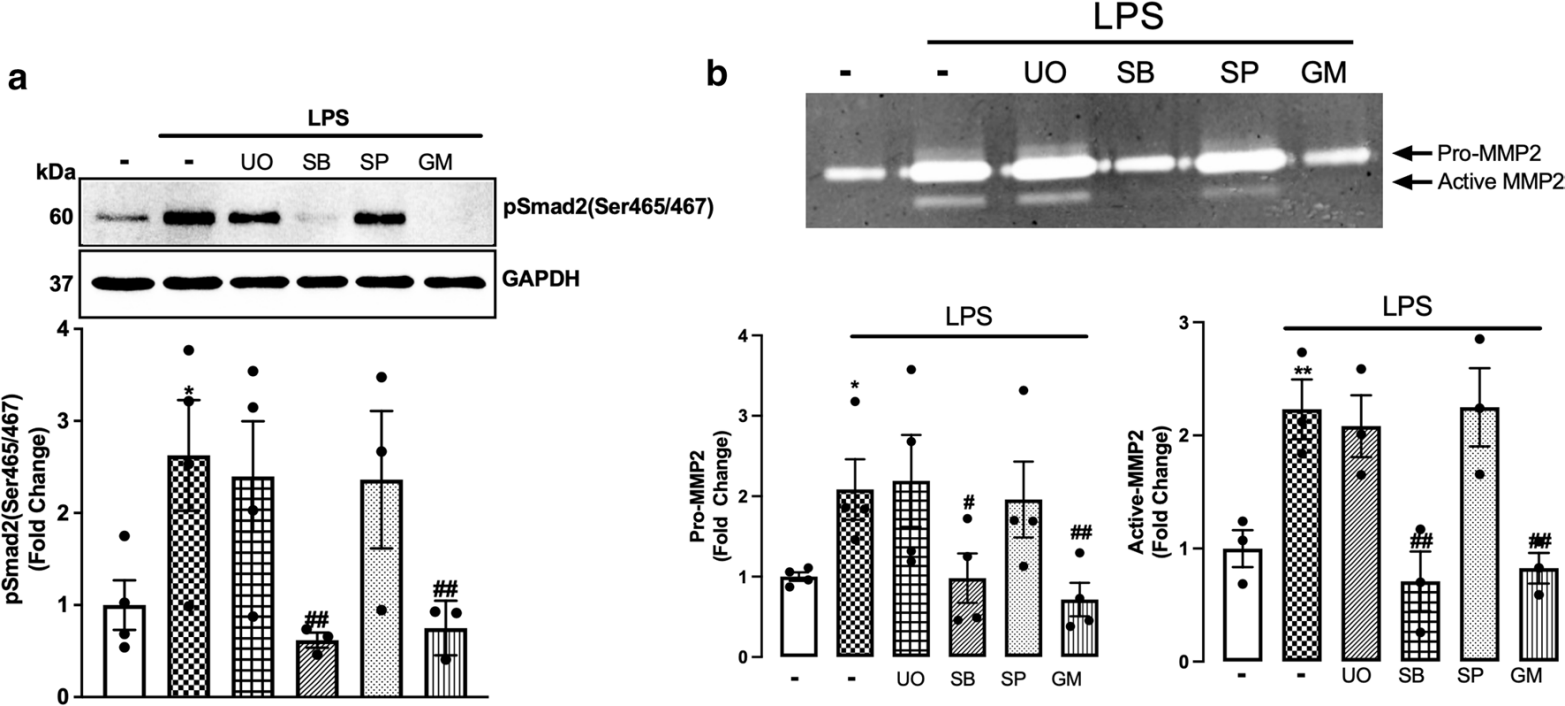
MAPK p38 is involved in TLR4 transactivation of TGFBR1 in VSMCs: HA-VSMCs were pre-incubated with U0126 (UO) (3 µM), SB202190 (SB) (3 µM), SP600125 (SP) (1 µM) and GM6001 (GM) **(a) **(10 µM) **(b) **(3 µM) for 30 min, followed by treatment with LPS (100 ng/mL) for 60 min. **(a)** Membranes were probed with antiphosphoSmad2 (Ser465/467) followed by HRP-labelled antirabbit IgG secondary, then stripped and probed with HRP-conjugated anti-GAPDH. **(b)** The gelatinase activity was assessed from spent media; transparent bands were detected against a background of Coomassie brilliant blue-stained gel. Histograms represent band density expressed as fold change compared to basal from four independent experiments. Statistical significance was determined using one-way ANOVA followed by LSD post hoc test, *p < 0.05 basal versus agonist and ##*p* < 0.01 agonist versus antagonist

TGF-β-mediated Smad2 carboxy terminal phosphorylation does not involve Erk, p38 and/or Jnk MAPKs [55]; rather, Smad2 carboxy terminal phosphorylation is a direct readout of TGFBR1 activation [56, 57]. In HSC-T6 cell lines, LPS-mediated Smad2 carboxy terminal phosphorylation involves Erk, p38 and Jnk-driven pathways [43]. Here, we investigated the role of Erk, p38 and Jnk in LPS-mediated Smad2 carboxy terminal phosphorylation utilising kinase-specific pharmacological inhibitors (Fig. 5a). LPS-mediated Smad2 phosphorylation was unaffected in the presence of Mek1/2 inhibitor, U0126, and Jnk inhibitor SP600125. In the presence of p38 inhibitor SB202190, LPS-mediated Smad2 phosphorylation was completely inhibited. MMP inhibitor GM6001 was used as a control, and completely inhibited LPS-stimulated phospho-Smad2.

We next investigated the role of MAPKs on MMP2 secretion in HA-VSMCs (Fig. 5b). LPS treatment increased the secretion of pro-MMP2 and active-MMP2 by twofold. MMP2 secretion was unaffected in the presence of U0126 and SP600125; however it was completely inhibited in the presence of p38 inhibitor SB202190. GM6001 was used as a control which showed complete inhibition of LPS-mediated MMP2 secretion. These data demonstrate that LPS via TLR4 transactivates the TGFBR1 to phosphorylate Smad2 via p38 and MMP2-dependent pathways.

### LPS stimulates PAI-1 mRNA expression in VSMCs via transactivation-dependent pathway

**Fig. 6:**
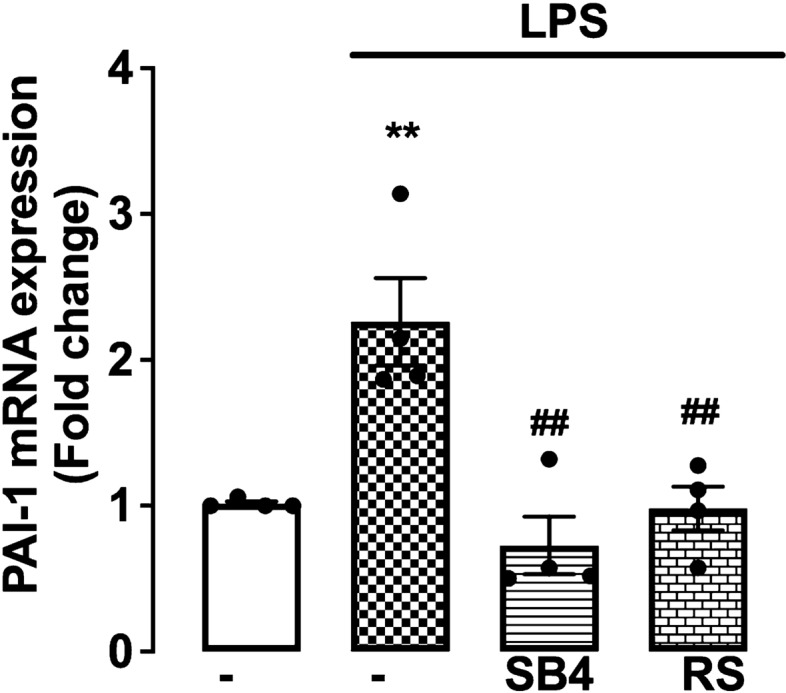
Role of LPS in PAI-1 gene expression. Human VSMCs cells were treated with SB431542 (3 µM) and LPS-RS (100 ng/mL) followed by treatment with LPS (100 ng/mL) for 8 h. Total RNA was extracted and the mRNA expression of PAI-1 was analysed using qRT-PCR. Results were presented as mean ± SEM from four separate experiments. Statistical significance was determined using one-way ANOVA followed by LSD post hoc test. **p < 0.01 basal versus agonist, ##*p* < 0.01 agonist versus antagonist

PAI‑1 plays a role in regulating the fibrinolytic system and is present in human atherosclerotic lesions. PAI-1 is markedly increased in patients with sepsis, leading to an increase in thrombus formation and severe organ dysfunction [58]. We investigated whether LPS-mediated PAI-1 expression occurs via transactivation-dependent pathways. HA-VSMCs were treated with LPS in the presence and absence of TGFBR inhibitor SB431542. We observed a 2.2-fold stimulation in PAI-1 mRNA expression (Fig. 6) that was completely inhibited in the presence of SB431542 and LPS-RS. These data demonstrate that LPS via TLR4 transactivates the TGFBR1 to stimulate PAI-1 mRNA expression in HA-VSMCs.

## Discussion

The engagement of receptor transactivation markedly expands the range of cellular responses attributable to the index cell surface, usually transmembrane, receptor. The current attributes of transactivation-dependent signalling encompass GPCR transactivation of kinase receptors [25, 49, 59–61]. Mechanistic studies of GPCR transactivation-dependent signalling define a concrete signalling pathway [23, 24, 32, 49]. LPS stimulates direct TGFBR1 intermediate phospho-Smad2 carboxy terminal via PI3K/Akt and MAPK-dependent pathways in HSC-T6 hepatic stellate cells [43]. We sought out to investigate the biological mechanisms involved in TLR4-mediated Smad2 phosphorylation. We demonstrated that in HA-VSMCs, treatment with LPS increased phospho-Smad2 carboxy terminal. LPS-mediated phospho-Smad2 carboxy terminal occurs via transactivation of the TGFBR1. We characterised the TLR4 transactivation of the TGFBR1 that occurs via p38 activation of MMP2, which cleaves and releases the TGF-β from the large latent TGF-β complex. LPS-mediated transactivation of the TGFBR1 upregulated the mRNA expression of PAI-1, highlighting the significance of this novel pathway in cardiovascular complications.

TLR4 recognised by LPS interacts with three extracellular proteins: LPS binding protein, CD14 and myeloid differentiation protein 2 (MD-2). Intracellularly TLRs recruit specific adapter molecules [7]. TLR4 signalling specifically signals via MyD88 and TRIF-dependent pathways to transduce a cellular response. We demonstrate, surprisingly, that LPS-mediated transactivation of the TGFBR1 occurs independently of the well-known MyD88 and TRIF-dependent pathways. LPS-RS competes with LPS for the same binding site on MD-2; utilising LPS-RS, we demonstrate that TLR4 transactivation of the TGFBR1 is conferred by extracellular proteins, specifically MD-2. Traditional activation of TLRs results in signalling via MyD88 and TRIF-dependent pathways; however, we demonstrate that TLR4 transactivation of the TGFBR1 occurs independently of these classical signalling pathways. TLR transactivation-dependent signalling represents a new signalling frontier because the identification that TLRs can transactivate other receptors greatly expands the range of signalling pathways and biological responses attributable to LPS.

In HSC-T6 hepatic stellate cells, LPS triggers the phosphorylation of Smad2 carboxy terminal that is inhibited by the TGFBR1 antagonist [43]. However, in skin fibroblasts, treatment with LPS augments the sensitivity of fibroblasts responding to TGF-β with a significant increase in Smad2 phosphorylation with co-treatment of LPS and TGF-β. The stimulation observed with co-treatment was completely lost in TLR4 silenced cells [62], which demonstrated a role for TLR4 in TGF-β-initiated signalling pathways. In contrast we demonstrate that in HA-VSMCs, TLR4-mediated phosphorylation of Smad2 occurs via the transactivation of the TGBFBR1. We also show that in TGF-β-treated cells, Smad2 phosphorylation is unaffected in the presence of competitive TLR4 antagonists LPS-RS. This demonstrates that this receptor-to-receptor communication is unidirectional in HA-VSMCs. In our studies, the cells were treated with LPS for 60 min to stimulate Smad2 phosphorylation; however, the skin fibroblasts’ [62] co-exposure of LPS and TGF-β was for 24 h. Exposure of cells to LPS for greater than 18 h results in the synthesis and release of TGF-β [63] which may have led to the augmented response observed in skin fibroblasts with co-treatment of LPS and TGF-β. Therefore, LPS via TLR4 transactivates the TGFBR1 to phosphorylate Smad2 in HA-VSMCs and HSC-T6 hepatic stellate cells [43] within a 60 min time frame.

Latent TGF-β remains sequestered as part of the LLC and a variety of mechanisms that can trigger release of the TGF-β from LLC [16]. Integrin-dependent activation of the LLC induces the release of latent TGFβ and activation of TGFBR1 in HA-VSMCs [19, 28]. Our data showed that pharmacological inhibition of ROCK signalling utilising ROCK inhibitor, Y27632, has no effect on LPS-mediated Smad2 carboxy terminal phosphorylation. However, in the presence of Y27632, thrombin-mediated Smad2 carboxy terminal phosphorylation was completely abolished. We show that GPCR agonists thrombin [19, 32, 49] and lysophosphatidic acid [24, 64] stimulate the phosphorylation of Smad2 carboxy terminal via Rho/ROCK-dependent activation of the TGFBR1. GPCR-dependent ROCK and integrin signalling leading to the transactivation of the TGFBR1 observed in VSMCs [25] was consistent with earlier observation in mouse lung epithelial cells [29], rat VSMCs [30] and airway smooth muscle cells [31]. We also show that endogenous ROS activation of the TGFBR2 occurs via ROCK-dependent pathways [50]. Given that LPS via TLR4 transactivates the TGFBR1 independent of Rho/ROCK pathways signifies that this is a completely new signalling pathway that warrants a full investigation.

MMPs play a crucial role in releasing and activating ECM‑sequestered growth and angiogenic factors, including TGF‑β [52]. MMP2 and MMP9 cleave the latent TGF-β binding peptide to facilitate the secretion of TGF‑β in foetal rat calvaria osteoblasts [65]. Glucose-induced phosphorylation of Smad3 in mouse embryonic fibroblasts and NRK‑52E cells depended on the proteolytic release of TGF-β from its complex [53]. Specifically, in mouse embryonic fibroblasts, the response was dependent on MMP2 and MMP9. Although several other MMPs have been identified to activate TGF-β signalling [66, 67], MMP2 and MMP9 are the most abundantly expressed in VSMCs. We demonstrated the specificity of the response by demonstrating that MMP2, but not MMP9 is involved in TLR4 transactivation of the TGFBR1. We further showed that the release of MMP2 is dependent on the MAPK, p38. This finding is consistent with several other observations that demonstrate that silencing of p38 [68, 69] or other MAPKs [70, 71] regulate the release and activity of MMP2.

In a genome-wide study, GPCR transactivation-dependent signalling accounted for approximately 50% of the total genes regulated by thrombin [27]. TGF-β plays a major role in mammalian homeostasis and is implicated in a diverse set of developmental disorders and diseases, including cancer, fibrosis, auto-immune and  CVDs [72, 73] including a potent stimulator of PAI-1 expression [17, 74]. To have a functional understanding of TLR transactivation of the TGFBR1, we measured the expression of PAI-1. The main physiological action of PAI-1 is to inhibit tissue plasminogen activator and urokinase and hence inhibit fibrinolysis. PAI-1 is upregulated in endothelial cells [75], mice lung and alveolar compartment [76] when exposed to LPS. Inhalation of LPS promoted coagulation and inhibited fibrinolysis in the lungs of healthy volunteers [77]. In the vasculature, PAI-1 has several roles in the pathogenesis of ischemic heart disease [78] including stabilising the fibrin matrix for migrating cells [79, 80]. We show that LPS-mediated PAI-1 expression occurs via transactivation of the TGFBR1 in human VSMCs. Higher levels of LPS are associated with increased risk of CVDs [7]. Our results demonstrated that TLR4 activated TGFBR1 to induce the expression of PAI-1. Therefore, TLR4 transactivation of TGFBR might play a potential role in the infection-induced development and progression of atherosclerosis.

We have demonstrated that LPS via TLR4 transactivates the TGFBR1 in VSMCs via p38 and MMP2-driven pathways to stimulate the expression of PAI-1. TLRs are first in line to recognise and respond to many exogenous invading substances (pathogens) and endogenous molecules. Our findings characterise a novel signalling pathway in the context of cell biology. Identifying TLR transactivation of the TGFBR1 may provide future insight into the detrimental implications of pathogens in pathophysiology.

## Data Availability

The data that support the findings of this study are available from the corresponding author upon reasonable request.
